# Early Social Enrichment Improves Social Motivation and Skills in a Monogenic Mouse Model of Autism, the* Oprm1* ^−/−^ Mouse

**DOI:** 10.1155/2016/5346161

**Published:** 2016-05-04

**Authors:** Luciana Garbugino, Eleonora Centofante, Francesca R. D'Amato

**Affiliations:** ^1^CNR, Institute of Cell Biology and Neurobiology, IRCCS Santa Lucia Foundation, Via del Fosso di Fiorano 64, 00143 Roma, Italy; ^2^F. M. Kirby Neurobiology Center, Department of Neurology, Boston Children's Hospital, 300 Longwood Avenue, Boston, MA 02115, USA

## Abstract

Environmental enrichment has been proven to have positive effects on both behavioral and physiological phenotypes in rodent models of mental and neurodevelopmental disorders. In this study, we used mice lacking the *µ*-opioid receptor gene (*Oprm1* ^−/−^), which has been shown to have deficits in social competence and communication, to assess the hypothesis that early enrichment can ameliorate sociability during development and adulthood. Due to the immaturity of sensory-motor capabilities of young pups, we chose as environmental stimulation a second lactating female, who provided extra maternal care and stimulation from birth. The results show that double mothering normalized the abnormal response to maternal separation in* Oprm1* ^−/−^ pups and increased social motivation in juveniles and adult knockout mice. Additionally, we observed that* Oprm1* ^−/−^ mice act as less attractive social partners than wild types, which suggests that social motivation can be modulated by the stimulus employed. This experiment supports previous findings suggesting that early social environmental stimulation has profound and long-term beneficial effects, encouraging the use of nonpharmacological interventions for the treatment of social defects in neurodevelopmental diseases.

## 1. Introduction

Modeling neuropsychiatric disorders in animals is an extremely challenging task because of the peculiarity of human symptoms, the lack of biomarkers, and the early state of knowledge of the relevant neurobiology and genetics. Defective social behaviors are among the symptoms shared by different neuropsychiatric disorders such as depression, schizophrenia, and autism. Social malfunctioning is the common trait of different mouse models of psychopathologies, deriving from genetic and environmental factors, and mainly consists in altered motor skills and sensory inputs and motivational defects.

Autism spectrum disorder (ASD) is a developmental disorder defined by impairments in social communication and social interactions and a restricted repertoire of activities and interests [1]. ASD is not associated with a single specific mutation, but several and different genetic abnormalities were found to be associated with the syndrome, frequently acting in combination and, possibly, interacting with environmental factors. Parallel to the investigation of the aetiology of ASD, emphasis has recently been placed on therapeutic approaches that can improve and/or delay the development of symptoms.

Pharmacological as well as environmental treatments have been applied and some of them proved to have positive outcomes, reducing the severity of some ASD symptoms. As an example of pharmacological treatments, the atypical antipsychotic risperidone reduces some symptoms in affected children [2, 3]. In mouse models as well, acute and chronic pharmacological treatments suggest improvement for some symptoms in young adult individuals, but long-lasting effects are rarely reported [4]. As far as behavioral therapies are concerned, it is well known that early treatments in ASD children show greater success in delaying and slowing down some symptoms.

It is difficult to model behavioral therapies in preclinical research, and environmental enrichment is the most common nonpharmacological treatment used in mouse models of several disorders (anxiety, depression, etc.). Environmental enrichment is usually applied from weaning onward and has almost always beneficial effects. Social and physical enrichment promote not only cognitive skills but also social interactions in several animal models [5–7]. Again, more pronounced and stable effects are associated with precocious exposure to the experimental environmental conditions [8]. Environmental enrichment usually involves housing conditions consisting of larger enclosures combined with more complex and variable physical and social environment. Exposing newborns to such an enriched environment, with them being deaf, blind, and almost immobile, could be useless because of the immaturity of their sensory-motor capabilities. Moreover, an early environment characterized by great variability could also represent a stressor for pups that need a secure (and stable) attachment basis to properly develop from an emotional point of view. The mother usually represents this stable basis: as a matter of fact, cross-fostering during the first days of life has a deleterious effect on pups' development [9–12].

The positive effects of environmental enrichment could reach the pups through the mother. Dams' behavior and physiology is affected by different housing conditions and this, in turn, can affect pups' development. Wild mice usually rear their pups in communal nests, where females, usually related, nurse and care for pups indiscriminately. To investigate the effects of such complex early environment, Branchi and coworkers performed a series of studies exploring the neurobiological and behavioral effects of being reared in communal nest, rather than in standard laboratory conditions, and reported more elaborated social competences in these mice [13–15]. Using a different experimental approach, we provided additional stimulation to both mothers and pups by housing the dam and her litter with a second (lactating or nonlactating) female allomother from birth onward. This socially enriched environment resulted in increased care for the pups and revealed positive outcome in terms of cognitive performance in outbred mice [16] and reduction of ASD symptoms in the fragile X syndrome mouse model [17].

The aim of this paper is to evaluate the possibility that the additional social stimulation, provided by the presence of a second lactating female from birth onward, can increase affiliative motivation in an animal model of social deficits. To this purpose, we used mice lacking the *µ*-opioid receptor gene (*Oprm1* ^−/−^) that have been proposed as a monogenic model of autism [18]. These animals show deficits in social behavior and communication from infancy onward, together with anatomical, neurochemical, and genetic landmarks of the disease [19–21]. The disruption of the *µ*-opioid signaling during development was able to induce deficits in infant-mother attachment, as well as in social interactions that persisted till adulthood. We hypothesized that the presence of a second female, which provides additional social stimulation, can improve social interactions in these *µ*-opioid knockout mice. For this purpose, we measured ultrasounds at postnatal day 8 (PND8) during isolation and sociability at weaning (PND28–30). Adult animals were then tested for emotionality, social recognition, and social preference. We expected that the early social enriched environment might affect social motivation and social-related behaviors in deficient *µ*-KO mice.

## 2. Materials and Methods

### 2.1. Subjects and Rearing Conditions

*µ*-opioid receptor knockout mice (*Oprm1* ^−/−^ and *µ*-KO) and their wild type controls (*Oprm1* ^*+/+*^ and WT), born from a colony raised in our animal facility, were used in this study. The generation of mice lacking *µ*-opioid receptors has been described and well characterized elsewhere [19, 22]. The colony was housed under constant temperature (20–22°C) and humidity conditions under a 12 h light/dark cycle with lights on at 7 am. Food and water were provided ad libitum. After weaning (PND28–30), animals were housed with same sex/genotype in standard mice cages containing 3–5 animals. WT and *µ*-KO subjects of these experiments were derived from homozygous breeding pairs.

Mating protocol consisted in housing two multiparous females (4-month-old) with one male of the same line for 15 days. After males were removed, according to reproductive status inferred from body weight increase (>35% from premating weight) and abdominal bulges presence (usually appearance on days 16-17 of gestation), females were assigned to one of the following experimental conditions: pregnant female housed alone (P) or two pregnant females housed together (P+P).

Around the expected day of partum, cages were inspected twice a day. After delivery, the number of pups in the P condition and the identity of the mother in the P+P cages were recorded. In the P+P condition, after at least 1 day (but no more than 5) elapsed between the two births, the younger litter was left in the cage while the older one was removed. P+P cages with females delivering on the same day or at a time interval superior to 5 days were discarded. In all conditions, cages with fewer than 4 pups were also discarded. The day of delivery was considered as PND0 and experimental conditions (P and P+P) were referred to hereafter as L and L+L since former pregnant (P) females were now lactating (L) females. Pups from the same litter were weaned, separated by sex, and housed in standard cages of 3–5 at PND28. Experimental animals derived from 7 *µ*-KO-L, 7 *µ*-KO-L+L, 7 WT-L, and 7 WT-L+L litters, with litter sizes between 4 and 8.

21–28-day-old male and female NMRI mice (Harlan) were used as stimulus partners in the Social Approach-Avoidance Test.

All experiments were conducted under license from the Italian Department of Health and in accordance with the Italian regulations on the use of animals for research (legislation DL 116/92 and 26/2014) and European guidelines on animal care.

### 2.2. Behavioral Tests during Development and at Adulthood

All experiments were carried out in enclosed rooms located in a sector outside the animal facility, to which animals were transferred approximately 1 h before the experiments started. The experimental rooms were kept under temperature and luminosity conditions equal to those of the animal facility (unless differently specified). With the exception of pups' ultrasonic vocalizations (USVs), all experimental sessions were video recorded and behavioral data were subsequently collected using The Observer (Noldus, Netherlands) or SMART (Panlab, Harvard Apparatus) software. As for the USVs, Avisoft technology (Avisoft Bioacoustics, Berlin, Germany) has been used to record and analyze the number of ultrasounds emitted by pups. Experiments were performed during the light phase between approximately 11 am and 4 pm. At the end of the test, cages were brought back to the breeding facility. Body weight of tested mice was measured at the end of the behavioral tests and the effects of genotype, rearing condition, and sex were evaluated during development (PND8), at weaning (PND28–30), and at adulthood (PND75–80).

#### 2.2.1. Ultrasonic Calls Emission

Pups' behavior was evaluated at PND8 by measuring USVs emitted during 5 minutes of isolation [21, 23]. After 1 h of acclimatization to the experimental room, the mother was removed and transferred into a clean cage, while pups were left in their home-cage, on a warm plate set at the temperature of 35°C to prevent cooling. No more than 4 pups/litter were employed. Pups were then individually placed into a beaker, containing own-cage (one male and one female) or clean bedding (one male and one female), and the vocalizations were recorded. Ultrasonic vocalizations were recorded using an UltraSoundGate Condenser Microphone (CM16, Avisoft Bioacoustics, Berlin, Germany) lowered 1 cm above the top of the isolation beaker containing the pup. The microphone was sensitive to frequencies of 15–180 kHz with a flat frequency response (±6 dB) between 25 and 140 kHz. It was connected via an UltraSoundGate USB Audio device to a personal computer, where acoustic data were recorded as wav files at 250,000 Hz in 16-bit format. Sound files were transferred to SasLab Pro (version 4.40; Avisoft Bioacoustics) for sonographic analysis and a fast Fourier transformation was conducted (512 FFT-length, 100% frame, Hamming window, and 75% time window overlap). Further details on this procedure, the device used, and the analysis of data can be found in our previous papers [16, 20]. The total number of ultrasounds emitted by each pup was analyzed by a 4-way ANOVA, the factors being genotype (*µ*-KO versus WT), early rearing condition (L versus L+L), bedding (clean versus home-cage), and sex (male versus females). For the sake of simplicity, a three-way ANOVA will follow in case of no effect of sex as independent variable, according to our previous results [20].

#### 2.2.2. Social Approach-Avoidance Test

Animals were tested immediately before weaning (PND28–30) in a gray Plexiglas rectangular box (60 × 40 × 24 cm) consisting of three same-size interconnected chambers. Two identical clear Plexiglas cylinders (8 cm in diameter) with multiple small holes were placed, one in each end chamber of the apparatus. During the habituation session (10 min), the mouse was placed in the central chamber and allowed to freely explore the whole apparatus. During the test session, a stimulus NMRI mouse, age/sex matched, was introduced into one cylinder (pseudorandomly chosen), whereas a white object was introduced into the other cylinder. Both 10 min sessions were recorded by a video camera and the time the subject mouse spent in each chamber and in proximity of each cylinder (2 cm: time close) was measured by a video-tracking system (SMART 1.1). After each test, the entire apparatus was carefully cleaned with 10% ethanol. Time spent in each chamber during habituation was scored to exclude any basal preference for one of the two lateral chambers. Time spent in proximity of each cylinder was analyzed by a 4-way ANOVA for repeated measures, the factors being genotype (*µ*-KO versus WT), rearing condition (L versus L+L), sex of the experimental subject (male versus female), and, as within factor, the stimulus in the cylinder (object versus mouse). For the sake of simplicity, a three-way ANOVA will follow in case of no effect of sex as independent variable, as expected by our previous results [20].

#### 2.2.3. Emotionality

Male and female mice were tested in the elevated plus maze at PND75–90 for emotionality. The elevated plus maze consisted of 2 open (5 cm wide and 30 cm long) and 2 closed arms (5 cm wide and 30 cm long, enclosed by a wall of 14 cm in height) arranged in a plus configuration, joined by a central square of 5 cm × 5 cm.

The apparatus was made of opaque Plexiglas and kept on a base 40 cm above the floor. Mice were exposed to a test of standard 5 min duration. At the beginning of the test, each mouse was placed individually in the center of the maze, with the head facing an open arm (the same for all mice). All tests were conducted between 13:00 h and 15:00 h and recorded by a video camera. The animals were initially accustomed to the experimental room for at least 1 hour before the experiment.

The time spent in the different arms of the apparatus was evaluated by automatic software analysis (Panlab SMART 1.1, Harvard Apparatus) and the total time spent on all four arms (Time Open + Time Closed: TO + TC), the number of entries (Entries Open: EO), and percentage of time spent in open arms (%TO = 100 × Time Open/(Time Open + Time Closed)) were used as behavioral indices of emotionality in three-way ANOVAs, the factors being genotype (*µ*-KO versus WT), rearing condition (L versus L+L), and sex of the subject (male versus female).

#### 2.2.4. Social Recognition (PND90–110)

The social recognition test has been used in previous studies [24, 25] as a social memory test to assess the ability of rodents to recognize animals they have been previously exposed to: mice show a characteristic decline in the time spent investigating a partner, with a full recovery following the introduction of a new conspecific. Subject mice were housed individually in clean cages for two days before test and served as residents. During test, which was performed in a soundproof cabin, a same sex/genotype, standard reared partner was introduced into the resident's cage. Partners were younger mice (PND45–70) housed in standard cages in groups of four/five mice. The partner remained in the resident's cage for one minute and the behavior of mice was video recorded. The partner was removed and returned into a clean home-cage for a 10-minute break. This procedure was repeated for a total of four identical sessions. During the fifth session, an unfamiliar, same sex/genotype and standard reared mouse partner from a new social cage was introduced and the behavior was video recorded for one minute. Video recordings were analyzed afterwards using The Observer software and the time spent by the resident male in social investigation (SI: sniffing and following the partner), as well as agonistic behaviors, was measured. A four-way ANOVA for repeated measures was applied to evaluate the effects of genotype (*µ*-KO versus WT), rearing condition (L versus L+L), sex (male versus female), and session (sessions A, B, C, D, and E) on the total amount of partner investigation during each 1 min session. For the sake of simplicity, separate three-way ANOVAs will follow in case of significant sex differences.

#### 2.2.5. Partner Preference (PND90–110)

This test was performed in a batch of adult animals naïve to the Social Approach-Avoidance Test. The same apparatus and the same procedure as in the Social Approach-Avoidance Test were used, with a variant during the test session: an unfamiliar conspecific was introduced into each cylinder. Social partners were age/sex matched with experimental subjects, but one was a wild type and the other a knockout animal. This would allow evaluating attractiveness of mice belonging to the two homozygous lines. The position of wild type and knockout partners within the apparatus was balanced within each group and was assigned independently of exploration time in each compartment during habituation. Both 10 min sessions (habituation and test) were recorded by a video camera and the time the subject mouse spent in each chamber and in proximity to each cylinder (2 cm: time close) was measured by a video-tracking system (SMART 1.1). After each test, the entire apparatus was carefully cleaned with 10% ethanol. Habituation preference scores were measured to evaluate a priori discrimination between lateral compartments. Preference for different line partners was evaluated by three-way ANOVAs, the factors being genotype (*µ*-KO versus WT), rearing condition (L versus L+L), and sex (male versus female). Partner preference was measured as % or time (sec) spent close to the cylinder containing the mouse with different genotype compared to that of the subject (preference score for different genotype = 100 × time close different genotype/(time close different genotype + time close same genotype)).

## 3. Results

### 3.1. Body Weight

Table 1 reports data on *µ*-KO and WT body weights measured in concomitance with behavioral tests. The first evaluation was conducted immediately after the USVs test, on PND8. The 3-way ANOVA revealed a strong effect of the rearing condition, with pups reared in the presence of their biological mother plus the second lactating female showing higher body weight (*p* < 0.001), independently of the genotype and sex. No interaction reached a significant effect. At weaning (PND28–30), the effect of the rearing condition on body weight disappeared and no significant main and interaction effects emerged from the ANOVA. Finally, mice were weighted once more (PND75–80) after the emotionality evaluation in the plus maze test. A strong sex effect emerged from body weight data, confirming that males were heavier than females, but no other main and interaction effects reached statistically significant levels.

### 3.2. Ultrasonic Calls Emission (PND8)

**Figure 1:**
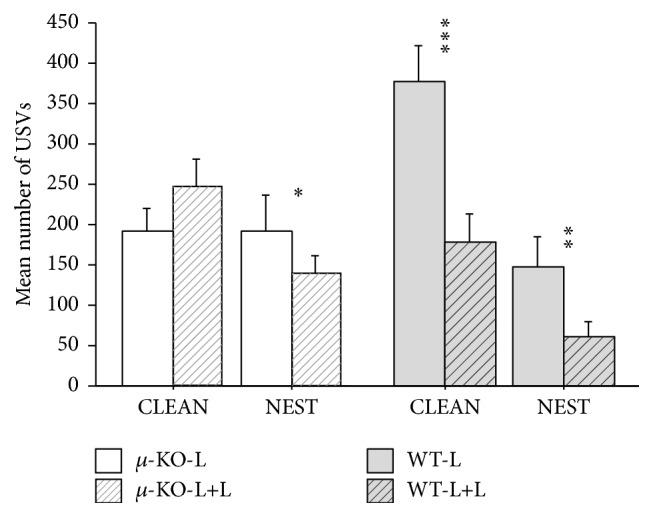
Mean number (+SEM) of ultrasonic vocalizations (USVs) emitted by 8-day-old pups of different experimental groups, when isolated in clean (CLEAN) or in their own home-cage (NEST) bedding for 5 min. *N*: 11–15 per group. *µ*-KO-L:* Oprm1* ^−/−^ pups reared by their mother [CLEAN: 9 males + 6 females; NEST: 8 males + 5 females]; *µ*-KO-L+L:* Oprm1* ^−/−^ pups reared by their mother plus a second lactating female [CLEAN: 4 males + 7 females; NEST: 6 males + 5 females]; WT-L:* Oprm1* ^*+/+*^ pups reared by their mother [CLEAN: 8 males + 6 females; NEST: 4 males + 8 females]; WT-L+L:* Oprm1* ^*+/+*^ pups reared by their mother plus a second lactating female [CLEAN: 7 males + 6 females; NEST: 6 males + 6 females]. Discrimination of clean versus nest bedding: ^*⁎*^ *p* < 0.05, ^*⁎⁎*^ *p* < 0.01, and ^*⁎⁎⁎*^ *p* < 0.001.

Isolated pups emitted ultrasonic calls as shown in Figure 1. According to our previous results [18], the 4-way ANOVA confirmed no significant main effect of the sex of the pup on USVs emission (male versus female: *F*_1/85_ = 2.16, ns). The successive three-way ANOVA indicated that the number of calls was strongly affected by rearing condition (L versus L+L: *F*_1/93_ = 20.10, *p* < 0.0001) and by experimental condition during isolation (clean versus home-cage bedding: *F*_1/93_ = 8.26, *p* < 0.01). In addition, genotype × rearing condition (*F*_1/93_ = 5.64, *p* < 0.05), genotype × bedding (*F*_1/93_ = 7.67, *p* < 0.01), and genotype × rearing condition × bedding reached significant effects (*F*_1/93_ = 4.64, *p* < 0.05). These results confirmed previous data indicating no differences between home-cage and clean bedding exposure in *µ*-KO pups and confirmed that these KO pups emitted fewer calls, in comparison with WT animals, when in clean bedding. Splitting up the analysis by genotype to specifically assess the effects of L+L rearing on pups' USVs, it emerged that early enrichment was not able to modify the amount of calls in *µ*-KO mice, when isolated in a clean environment (*µ*-KO clean: *F*_1/24_ = 0.01, ns), but reduced USVs in all other groups (*µ*-KO nest: *F*_1/22_ = 4.91, *p* < 0.05; WT clean: *F*_1/25_ = 14.89, *p* < 0.001; WT nest: *F*_1/22_ = 9.52, *p* < 0.01).

### 3.3. Social Approach-Avoidance versus NMRI (PND28–30)

**Figure 2:**
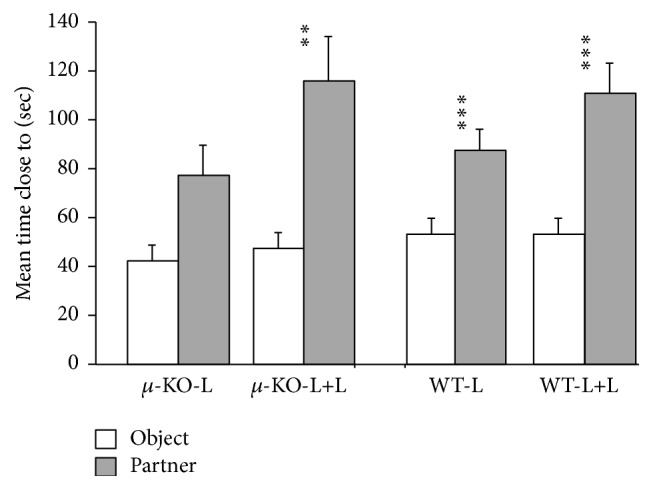
Mean time (+SEM) spent by juvenile male and female PND28 mice close to an object or to a same age/sex NMRI outbred partner in the Social Approach-Avoidance Test. *N*: 14–18 per group. *µ*-KO-L:* Oprm1* ^−/−^ pups reared by their mother [8 males + 6 females]; *µ*-KO-L+L:* Oprm1* ^−/−^ pups reared by their mother plus a second lactating female [8 males + 10 females]; WT-L:* Oprm1* ^*+/+*^ pups reared by their mother [8 males + 9 females]; WT-L+L:* Oprm1* ^*+/+*^ pups reared by their mother plus a second lactating female [8 males + 8 females]. Preference of partner versus object: ^*⁎⁎*^ *p* < 0.01, ^*⁎⁎⁎*^ *p* < 0.005.

Male and female adolescent mice did not differ for time spent close to the object or social stimulus contained in cylinders (*F*_1/57_ = 0.53, ns). The subsequent three-way ANOVA confirmed that all young mice spent more time close to conspecific rather than the object (*F*_1/61_ = 31.58, *p* < 0.0001). However, a significant effect of the rearing condition per se (*F*_1/61_ = 5.21, *p* < 0.05, Figure 2) emerged, together with a tendency towards a rearing condition × genotype (*F*_1/61_ = 3.47, *p* = 0.07) and rearing condition × stimulus effect (*F*_1/61_ = 3.57, *p* = 0.06). Considering together male and female performance, all experimental groups, but not *µ*-KO-L (*F*_1/13_ = 2.27, ns), spent more time close to the conspecific rather than the object (*µ*-KO-L+L: *F*_1/17_ = 8.79, *p* < 0.01; WT-L: *F*_1/16_ = 11.5, *p* < 0.001; WT-L+L: *F*_1/15_ = 15.86, *p* < 0.01).

### 3.4. Emotionality (PND75–90)

**Figure 3:**
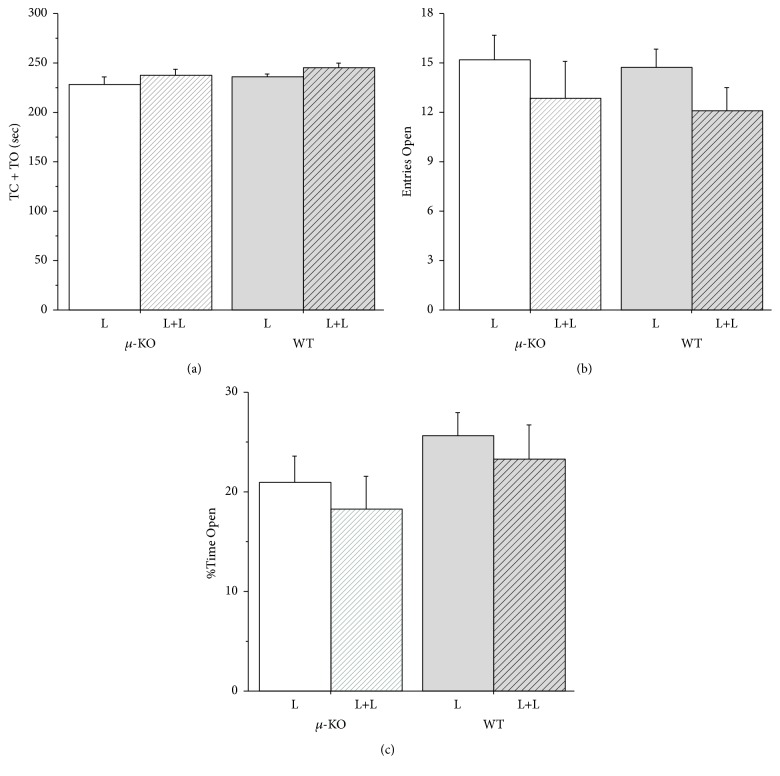
Adult mice emotionality measure in the plus maze apparatus: (a) Time Open + Time Closed, (b) number of entries in the open arms, and (c) percentage of time spent in the open arms. Data are presented as mean (+SEM). *N*: 11–20 per group. *µ*-KO-L:* Oprm1* ^−/−^ pups reared by their mother [9 males + 9 females]; *µ*-KO-L+L:* Oprm1* ^−/−^ pups reared by their mother plus a second lactating female [6 males + 5 females]; WT-L:* Oprm1* ^*+/+*^ pups reared by their mother [8 males + 12 females]; WT-L+L:* Oprm1* ^*+/+*^ pups reared by their mother plus a second lactating female [11 males + 6 females].

Emotionality of adult WT and *µ*-KO mice was measured in the plus maze apparatus (Figure 3). No significant effect either of genotype, rearing condition, or sex was observed for the three parameters considered (TC + TO: *F*_1/58_ = 1.98, ns; rearing condition: *F*_1/58_ = 2.59, ns; sex: *F*_1/58_ = 0.001, ns; EO: genotype: *F*_1/58_ = 0.37, ns; rearing condition: *F*_1/58_ = 3.35, ns; sex: *F*_1/58_ = 1.95, ns; %TO: genotype: *F*_1/58_ = 2.46, ns; rearing condition: *F*_1/58_ = 1.01, ns; sex: *F*_1/58_ = 2.58, ns). In addition, no significant interaction effect was detected.

### 3.5. Social Recognition (PND90–110)

**Figure 4:**
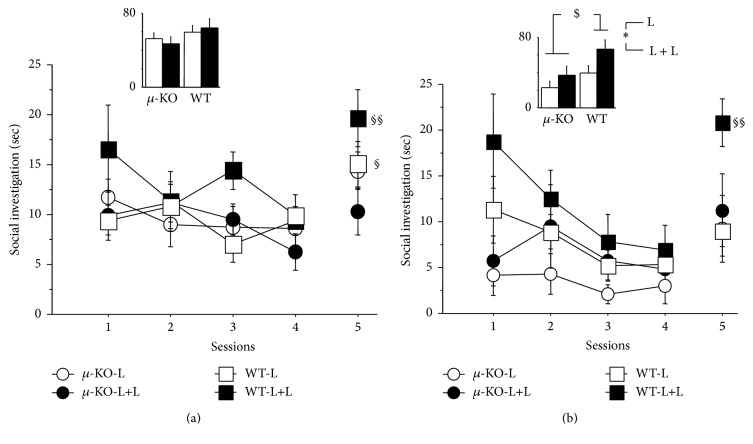
Mean time (+SEM) spent by female (a) and male (b) mice in social investigation of a younger same sex/genotype/standard reared intruder mouse during 5 consecutive 1 min social interaction sessions (line graph). The same intruder was used during the first 4 sessions, while an unknown one (same characteristics) was introduced in the fifth session. Histograms represent the total amount of investigation shown during the five sessions by different experimental groups. *N*: 8–15 per group. *µ*-KO-L:* Oprm1* ^−/−^ pups reared by their mother [10 males + 15 females]; *µ*-KO-L+L:* Oprm1* ^−/−^ pups reared by their mother plus a second lactating female [9 males + 11 females]; WT-L:* Oprm1* ^*+/+*^ pups reared by their mother [10 males + 15 females]; WT-L+L:* Oprm1* ^*+/+*^ pups reared by their mother plus a second lactating female [8 males + 11 females]. Genotype difference: ^$^ *p* < 0.05; rearing condition difference: ^*⁎*^ *p* < 0.05; recognition of new partner (session 4 versus 5 and paired *t*-test): ^§^ *p* < 0.05 (*µ*-KO-L and WT-L females) and ^§§^ *p* < 0.01 (*µ*-KO-L+L males and females).

Two relevant pieces of information emerge from data collected in this test: the first one refers to the interest in a social partner (total amount of social investigation) and the second one to the capability to recognize the same partner (decrease in investigation from session 1 to 4), from an unknown individual (session 4 versus 5). The general analysis indicated that all mice were able to recognize partners' familiarity, during repeated exposures, according to difference/reduction in time spent investigating it during different sessions (*F*_4/324_ = 13.97, *p* < 0.0001). However, significant genotype (*F*_1/81_ = 8.78, *p* < 0.01), sex (*F*_1/81_ = 5.68, *p* < 0.01), and genotype × rearing condition × session effects were detected (*F*_4/324_ = 4.01, *p* < 0.01). We analyzed separately these data in female and male mice, as shown in Figures 4(a) and 4(b), respectively. When considering the total amount of time spent investigating the partners in the 5 consecutive sessions (Figure 4(a), bar graph), no significant differences emerged for females according to the genotype (*F*_1/48_ = 2.43, ns), rearing conditions (*F*_1/48_ = 0.01, ns), and their interaction (*F*_1/48_ = 0.38, ns). Females (Figure 4(a), line graph) showed a significant time (*F*_4/192_ = 7.21, *p* < 0.0001) and a general interaction effect only (genotype × rearing condition × session: *F*_4/192_ = 4.09, *p* < 0.01), suggesting recognition of the unknown individual in the fifth session, an effect statistically significant in *µ*-KO-L and in WT-L and WT-L+L animals. As for males (Figure 4(b), bar graph), *µ*-KO spent less total time in social investigation in comparison with WT mice (*F*_1/33_ = 6.57, *p* < 0.05) and animals reared by double mothering scored higher than those reared in standard condition (*F*_1/33_ = 5.10, *p* < 0.05), with no significant interaction between factors (*F*_1/33_ = 0.50, ns). Investigating partner's recognition capability (Figure 4(b), line graph), significant genotype (*F*_1/132_ = 5.38, *p* < 0.05), rearing condition (*F*_1/33_ = 4.10, *p* = 0.05), and session effects emerged (*F*_4/132_ = 6.54, *p* < 0.0001), stressing very low levels of social investigation in *µ*-KO mice, and rearing condition (L+L versus L) increasing this behavior in both genotypes. Only WT males reared by two lactating females were able to discriminate an unknown from a familiar mouse (session 4 versus 5). Only 7 out of 89 residents showed some agonistic behavior (total 5-session score between 3.3 and 18.3 sec).

### 3.6. Partner Preference (PND90–110)

**Figure 5:**
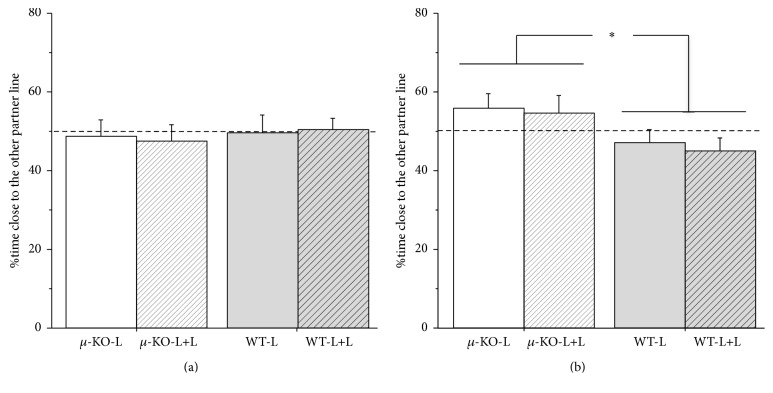
Mean (+SEM) percentage of time spent by adult females (a) and males (b) close to a same sex partner of the other homozygous line in a modified version of the Approach-Avoidance Test, where a *µ*-KO and a WT partner mouse were simultaneously presented to the experimental subject. The dotted line represents chance level (50%). *N*: 12–16 per group. *µ*-KO-L:* Oprm1* ^−/−^ pups reared by their mother [16 males + 16 females]; *µ*-KO-L+L:* Oprm1* ^−/−^ pups reared by their mother plus a second lactating female [15 males + 15 females]; WT-L:* Oprm1* ^*+/+*^ pups reared by their mother [16 males + 13 females]; WT-L+L:* Oprm1* ^*+/+*^ pups reared by their mother plus a second lactating female [15 males + 12 females]. Difference due to genotype ^*⁎*^ *p* < 0.05.

Adult *µ*-KO and WT mice were tested in a modified version of the Approach-Avoidance Test where the subject was simultaneously exposed to mice with different genotypes. First of all, the total time spent close to partners (data not shown) was not affected by genotype (*F*_1/110_ = 0.15, ns) and sex of the subject (*F*_1/110_ = 2.36, ns) but was slightly reduced in animals reared by two females (*F*_1/110_ = 4.31, *p* < 0.05). We then tested whether there was a difference in partner preference (same line versus different line) according to the genotype and early experience of the subject. In Figures 5(a) and 5(b), the %preferences for WT in *µ*-KO and for *µ*-KO in WT female and male subjects are reported, respectively. All female groups, independently of genotype (*F*_1/52_ = 0.51, ns) and rearing condition (*F*_1/52_ = 0.25, ns), showed similar interest towards their female partners, whether the latter were *µ*-KO or WT. Males behaved differently. *µ*-KO showed preference, whereas WT males showed avoidance of males of the alien line (*F*_1/58_ = 7.17, *p* < 0.01), indicating a generalized avoidance of all subjects towards *µ*-KO male partners. No other significant effects emerged.

## 4. Discussion

Many studies have shown that environmental enrichment, once applied to animals previously reared in standard husbandry, is able to revert and/or prevent pathological conditions resulting from genetic, environmental, and pharmacological insults [26]. This experimental condition, highly variable across studies, includes generally sensory-motor stimulation that provides the animal with increased opportunities for physical exercise, learning experiences, and social interactions. Whether this early environmental enrichment (EEE) condition represents enrichment or rather, a reduction of deprivation condition, is not an issue considered here. Juvenile animals may utterly benefit from these additional stimulations especially when provided during the early postnatal life, a period of development characterized by neural plasticity. In this case, EEE may represent a possible alternative inexpensive treatment.

The mother represents the main component of the environment of an infant laboratory mouse and, in order to enhance stimulation during early life, we manipulated mother-infant interactions. We have already shown that this form of early environmental enrichment, consisting in housing pups with an additional lactating or nonlactating female from birth until weaning, exerts long-lasting beneficial effects on brain and behavior in outbred mice [16]. A similar approach has also been used in the Fmr1-KO mice and significant long-term beneficial effects on the pathological fragile X syndrome (FXS) phenotype have been detected [17]. Specifically, this rearing condition rescued Fmr1-KO adult mice deficits, namely, hyperactivity, social interactions, and cognitive deficits. In addition, early social enrichment also eliminated the abnormalities shown by adult Fmr1-KO mice in the morphology of hippocampal and amygdala dendritic spines. Importantly, this rearing condition did not induce neurobehavioral changes in WT mice, thus supporting specific effects on FXS-like pathology.

The aim of the present study was to evaluate whether being reared from birth in a socially enriched environment could also affect social motivation and behavior in a mouse model of social deficit: the *µ*-opioid knockout mice. These knockout animals have already been characterized for their deficits in social behavior from infancy onward and for this reason they seem to be the ideal candidates for this study. Firstly, we will discuss the results observed in WT animals to ascertain the impact of this early social enrichment on the social phenotype; then, we will assess whether this rearing condition is able to rescue the social deficits in this monogenic mouse model of autism [18].

We did not compare our dams' molecular and behavioral profile with that of females housed in EEE [27], but the presence of a second female in the breeding cage could represent a condition of environmental enrichment for the mother, as well as for the pups. As for pups, as a result of higher parental care, they might weigh more during development (as reported here) and at adulthood [16, 17]. In addition, tactile stimulation as well as olfactory and gustatory information from the adoptive female should expand the range of stimuli pups were exposed to from birth. Data presented here indicate that control pups reared by two lactating females (WT-L+L) vocalized less than standard reared wild type pups (WT-L) (Figure 1) but did not differ from them in their interest in conspecifics (Figure 2), emotionality (Figure 3), and partner preference (Figure 5). Double mothering increased social investigation in adult male WT mice, improving social competence (social recognition, Figure 4), an effect already reported in communal reared outbred mice [15].

In the present study, all animals in the breeding cages shared the same genotype: *µ*-KO and WT mice were maintained in homolines and the allomother had the same genotype as the mother-infant dyad. This could have restricted the variability of new stimuli supplied by the allomother to developing pups, since females with the same genotype may share similar physical and behavioral characteristics but should nevertheless have increased maternal cues, adding together stimuli from the two mothers. This strategy was selected to provide a quantitatively more significant stimulation to *µ*-KO pups to facilitate the development of infant attachment bond to this “super-mother,” possibly by recruiting alternative neurobiological systems participating in the reward circuit. High levels of pup grooming, for example, should result in an increase of OTR expression in the MPOA [28, 29] and stroking behavior in adult rats activates hypothalamic oxytocin neurons [30]. The higher amount of oxytocin released as a consequence of higher amount of dams' stimulation may activate the dopaminergic reward pathways in response to maternal cues, also in the absence of a functional *µ*-opioid system.

Data presented here partially support our hypothesis: being reared in a socially enriched environment seems to be able to rescue social motivational deficits shown by *µ*-opioid receptor knockout mice [20]. In fact, *µ*-KO pups reared by two lactating females showed (1) differential USVs emission, according to test context, and (2) preference for a social stimulus versus an object at weaning, as WT mice.

Isolated pups usually emit ultrasonic vocalizations, and the presence of familiar nest odors leads to a reduction of these calls [21, 31–34]. The low and similar number of USVs emitted by *µ*-KO pups in clean and nest condition suggested the following: (1) no calming effect of familiar cues and (2) lack of attachment behavior in these mutant pups [21]. Data presented here indicate that the emotional response to separation/isolation can be modified in *µ*-KO pups by rearing condition. While the effects of strongly aversive rearing conditions on pups' emotional responses were already known, the effects of an enriched/positive environment have more rarely been reported.

Double mothering could have quantitatively and qualitatively increased the amount of olfactory/tactile/gustatory and nursing stimulation, accelerating maturation processes and/or increasing stress thresholds. This hypothesis seems acceptable for WT animals, which showed a generalized tendency to vocalize less. The same occurred in *µ*-KO-L+L pups, which behaved as their WT-L+L controls, reducing isolation-induced USV emission in the presence of their home-cage odor. These results suggest that double mothering in *µ*-KO mice, by accelerating maturation processes and/or by providing additional maternal stimulation, restored the differential emotional response to mothers' cues presence. Home-cage bedding had a calming effect on *µ*-KO-L+L, whatever the contribution of the mother(s) and/or littermates in this process. The rescue of social motivation by early social enrichment emerged also from the social performance in juveniles (Figure 2). *µ*-KO-L+L mice behaved as WT animals, showing preference for the social versus the inanimate stimulus. It should be stressed that in this case the partner was an NMRI mouse, an albino conspecific with characteristics completely different and new for both WT and *µ*-KO subjects.

Contrary to what was expected on the basis of data from the literature that linked the amount of maternal care received with the HPA axis activity [35], rearing conditions did not modify emotionality in our mice, at least when measured in the plus maze apparatus. Similar results have already been reported in our previous studies [16], supporting the idea that it is not simply the amount of licking and grooming that epigenetically modulates gene transcription.

Social skills in adult animals were assessed in the social recognition test. This test allows evaluating, not only the amount of social interaction with unknown conspecifics, but also the mouse's ability to discriminate between known and unknown intruders. Females did not differ either for genotype or for early social environment in the total amount of social investigation towards the female intruder and generally recognized the new partner in the fifth/last social session, when the unknown subject was presented (but not the *µ*-KO-L+L group). As for males, the presence of the two lactating females increased social investigation in WT as well as in *µ*-KO mice, the latter showing very low interest in conspecifics in standard condition, confirming a reduced social motivation to interact with peers [20].

Finally, we wondered whether the reduced sociability of *µ*-KO animals might depend on their partners' characteristics. The results of the social preference test indicated that both WT and *µ*-KO mice preferred WT partners. This suggests that social performance of *µ*-KO mice could have been affected by the characteristics of their *µ*-KO partners in social tests (but not in the Approach-Avoidance Test, where an NMRI neutral animal was presented). The relevance of this result lays on the fact that partner's characteristics, and whether these match the experimental subject's preferences, are usually not considered. Sex, strain, food eaten, social rank, reproductive condition, and others are some of the characteristics that can affect the attractiveness of a partner for one particular subject [36]. It is possible that, in the presence of a different partner, we could improve social performance of “antisocial” animals as well, or we could improve sociability in these individuals through repeated interactions with preferred partners. This aspect has been investigated by Crawley's group in BTBR mice, a mouse strain characterized as an ASD model because of its poor sociability and repetitive social behaviors. Interestingly, they found no deficit in sensory inputs in the BTBR mouse but an improving effect of cohousing after weaning with the “social” C57/B6 mice [37].

Wild type and *μ*-KO mice that were used for the present experiments were derived from homozygous breeding pairs. If we consider the copresence of two *μ*-KO mothers to be equivalent to the caring work done by one wild type mother, if *μ*-KO mothers are to show any maternal care deficit, then we might not exclude the hypothesis that the early social enrichment has cured the phenotype of the homozygous* Oprm1*-deficient mother rather than that of the pup and juvenile −/− mice. However, we did not find differences in maternal care behavior between *μ*-KO and WT mothers in our previous study [20]. Therefore, we are prone to consider the effects of the social enrichment acting directly on the pups, although we do not exclude that also the mothers might have benefited from it. In future experiments it remains to be determined whether the present results can be replicated with pups derived from heterozygous mothers.

## 5. Conclusions

This experiment suggests that social environmental enrichment during early postnatal life can reduce stress during development and improve sociability in social defective subjects. We are not aware whether pups benefit directly from additional warmth, olfactory, tactile, or nutritional stimulation provided by the second dam in the cage or whether they received indirect benefits through their mother's more relaxed state. To this purpose, particular attention has been devoted in this study to reducing conflict between the dams, housing them together, from mating onward. Whatever the causal mechanisms, *µ*-KO mice reared by their mother plus an additional lactating female showed increased social motivation from early age to adulthood. These results may encourage the investigation of the causal mechanisms underlying the rescue of social behavior we reported, in order to promote useful therapeutic interventions for all those developmental psychopathologies characterized by social malfunctioning.

## Figures and Tables

**Table 1:** Mean (±SEM) body weight (gr.) of mice (males and females) belonging to the four experimental groups.

Experimental groups	*Oprm1* ^−/−^		*Oprm1* ^+/+^		ANOVA		
	L	L+L	L	L+L	*F*_1/68_ G	*F*_1/68_ RC	*F*_1/68_ S
Day 8							
Males	4.68 ± 0.15 [9]	5.99 ± 0.23 [8]	4.60 ± 0.24 [13]	6.49 ± 0.10 [9]	0.78	152.02^*∗∗∗*^	0.14
Females	4.68 ± 0.20 [7]	6.31 ± 0.17 [10]	4.65 ± 0.17 [11]	6.40 ± 0.16 [9]			

					ANOVA		
					*F*_1/56_ G	*F*_1/56_ E	*F*_1/56_ S

Days 28–30							
Males	16.32 ± 0.48 [8]	14.08 ± 0.51 [10]	14.86 ± 0.79 [8]	15.03 ± 0.89 [8]	1.69	1.41	0.21
Females	15.64 ± 0.44 [6]	15.06 ± 0.56 [8]	14.17 ± 0.29 [8]	14.54 ± 0.66 [8]			

					ANOVA		
					*F*_1/66_ G	*F*_1/66_ E	*F*_1/66_ S

Days 75–80							
Males	27.17 ± 0.59 [11]	28.06 ± 0.41 [5]	25.75 ± 0.87 [14]	26.68 ± 0.54 [6]	0.68	0.53	90.42^*∗∗*^
Females	21.36 ± 0.31 [9]	21.75 ± 0.30 [6]	22.39 ± 0.82 [12]	21.74 ± 0.53 [11]			

G: genotype; RC: rearing condition; S: sex. ^*∗∗*^ *p* < 0.001, ^*∗∗∗*^ *p* < 0.0001.

Sample sizes are reported in brackets.
